# Predicting bacterial resistance from whole-genome sequences using *k*-mers and stability selection

**DOI:** 10.1186/s12859-018-2403-z

**Published:** 2018-10-17

**Authors:** Pierre Mahé, Maud Tournoud

**Affiliations:** bioMérieux, Chemin de l’Orme, Marcy l’Etoile, 69280 France

**Keywords:** Genotype-phenotype, Feature selection, *k*-mers, Lasso

## Abstract

**Background:**

Several studies demonstrated the feasibility of predicting bacterial antibiotic resistance phenotypes from whole-genome sequences, the prediction process usually amounting to detecting the presence of genes involved in antibiotic resistance mechanisms, or of specific mutations, previously identified from a training panel of strains, within these genes. We address the problem from the supervised statistical learning perspective, not relying on prior information about such resistance factors. We rely on a *k*-mer based genotyping scheme and a logistic regression model, thereby combining several *k*-mers into a probabilistic model. To identify a small yet predictive set of *k*-mers, we rely on the stability selection approach (Meinshausen et al., J R Stat Soc Ser B 72:417–73, 2010), that consists in penalizing logistic regression models with a Lasso penalty, coupled with extensive resampling procedures.

**Results:**

Using public datasets, we applied the resulting classifiers to two bacterial species and achieved predictive performance equivalent to state of the art. The models are extremely sparse, involving 1 to 8 *k*-mers per antibiotic, hence are remarkably easy and fast to evaluate on new genomes (from raw reads to assemblies).

**Conclusion:**

Our proof of concept therefore demonstrates that stability selection is a powerful approach to investigate bacterial genotype-phenotype relationships.

**Electronic supplementary material:**

The online version of this article (10.1186/s12859-018-2403-z) contains supplementary material, which is available to authorized users.

## Background

Recent advances in Next-Generation Sequencing (NGS) technologies provided new tools to sequence large amounts of DNA at a reasonable cost and in a limited period [1]. This technological breakthrough is expected to significantly modify the landscape and practices in the field of clinical microbiology. Microorganisms can now be characterized with unprecedented resolution, which can have a significant impact for both research and diagnostics purposes (see e.g., [2, 3]). In terms of diagnostics, NGS indeed holds the promise of addressing, in a single experiment, the main questions of clinical interest: identifying an isolate and determining its antibiotic resistance and virulence profile [4]. The genetic bases of antibiotic resistance and virulence remain partly unknown for most bacterial species, and it is still on open question whether the resistance or virulence of a microorganism can be inferred from its genome only. A recent study showed for example that an isogenic bacterial population exhibited heterogeneity in drug susceptibility due to random partitioning of efflux-pumps during cellular division [5].

Nevertheless, several works have demonstrated the feasibility of genotypic approaches for detection of antibiotic resistance, where a good concordance has been observed between resistance phenotypes predicted from microorganisms genomes, and their reference phenotypes, determined experimentally by assessing their ability to grow in the presence of antibiotics. The genetic bases of antibiotic resistances are for instance well known for *Staphylococcus aureus* and *Mycobacterium tuberculosis*, and accurate predictions could be achieved for these species by simply detecting specific genetic resistance determinants [6–11]. This strategy is notably implemented in well-established tools like Mykrobe [7] and TBProfiler [9], that leverage for instance catalogs of more than 200 and 1300 mutations, respectively, to predict resistance of *M. tuberculosis* to 9 and 10 antibiotics or antibiotic families. While this approach proved to be effective for these relatively clonal species, it may suffer from two limitations if transposed to other bacterial species and/or drugs. The first limitation is that it intrinsically relies on prior knowledge of resistance determinants, which may not be available for all species. Non-coding regions are not explored either, which could be beneficial even for *M. tuberculosis*, for which resistance is mostly due to the presence and accumulation of mutations and indels within a limited number of core genes [12–14]. Secondly, resistance prediction rules typically rely on the presence of at least one resistance determinant, whereas it may be beneficial to combine several ones in a common prediction model to address complex multi-factorial resistance mechanisms, or to model the accumulation of several mutations eventually leading to resistance [12, 15]. With these two limitations in mind, we address the problem from the supervised learning perspective and build multi-factorial prediction rules from a panel of strains, for which whole-genome sequences and resistance phenotypes are available, without relying on any prior knowledge about the resistance determinants. We adopt a systematic *k*-mer based strain genotyping scheme, where any possible *k*-mer is a candidate determinant, and rely on the logistic regression model to combine several *k*-mers into a probabilistic prediction rule.

Genotyping strains with *k*-mers, where every sequence of length *k* found in a genome is a putative resistance determinant, allows to circumvent knowing the genes involved in antibiotic resistance. This approach offers the additional benefits of being alignment-free and able to capture various types of genetic determinants, like the presence of genes, as well as Single-Nucleotide Polymorphisms (SNPs) and indels that can be located in coding or non-coding regions [16]. Such *k*-mer based representations are therefore increasingly popular in this context, for both genome-wide association studies [16, 17] and predictive modelling [18, 19]. The probabilistic framework offered by the logistic regression model is also appealing. First, it naturally combines several genomic determinants in a global predictive model with weights modulating their respective effects, hence quantifying their relative predictive power and reflecting the fact that they can be associated with different levels of resistance [12]. It also provides a probabilistic prediction, which allows to quantify the confidence the user can have in the results provided.

Our approach is closely related to [18, 19], who recently relied on machine learning approaches to predict categorical antibiotic resistance phenotypes from *k*-mers. The resulting prediction rules are based on the detection of possibly multiple *k*-mers, which are automatically selected by the algorithm and are respectively combined by means of logical operations (conjuctions or disjunctions) or a linear combination. Likewise, [20] recently relied on a standard linear regression model to predict the Minimum Inhibitory Concentration (MIC) from candidate mutations found in pre-defined genes, and [11] explored several machine learning strategies to predict *M. tuberculosis* resistance from a pre-defined list of SNPs, with promising results. By combining *k*-mers and logistic regression, we therefore aim to bridge the gap between these two approaches, hence to build prediction models allowing to combine several genomic determinants within a versatile probabilistic framework, without relying on the prior knowledge of the underlying resistance mechanisms.

For the sake of interpretability and computational efficiency of the prediction, we have the utmost interest in building concise models, involving as few genetic determinants as possible. From the statistical learning perspective, the challenge is to identify a small yet predictive set of *k*-mers from a very large number of redundant and correlated ones (several 100.000’s). We rely for this purpose on the so-called stability selection approach [21], that consists in penalizing the logistic regression model with the sparsity-promoting Lasso penalty, within an extensive resampling procedure. We present a proof of concept of this approach for *M. tuberculosis* and *S. aureus* using existing datasets [7, 19]. Our main contribution is to demonstrate that stability selection is a very efficient strategy in this context, leading to robust and extremely sparse signatures of resistance. The empirical results obtained allow furthermore to differentiate between the association and prediction perspectives on antibiotic resistance in bacteria, and suggest several promising leads for further work.

## Methods

### Strain genotyping with *k*-mers

To genotype a training panel of *n* assembled bacterial genomes using *k*-mers, we first build a large matrix encoding the presence or absence of all *k*-mers of length *k*=31 within each strain, using the Ray software [22]. Considering *k*-mers of length 31 is a safe default choice in this context, offering in general a good trade-off between sequence specificity and computational efficiency [18, 19]. Since *k*-mers occurring in too few or too many strains have a limited predictive power, we then discard *k*-mers found in one or two strains only, or in all the strains but one or two. Finally, since this *k*-mer based representation is highly redundant [18, 19], we define sets of equivalent *k*-mers from *k*-mers having the same presence/absence profile in all the strains. For the sake of model building, we can only keep one of them, which in practice dramatically reduces the number of *k*-mers to consider. We keep track of these sets of equivalent *k*-mers in order to better interpret the model obtained and carry out the predictions, as discussed in “Prediction from genome assembly or sequencing reads” and “Model interpretation” sections. Equivalent *k*-mers typically correspond to contiguous stretches of sequences conserved across strains, but can also correspond to non-contiguous stretches in total Linkage Disequilibrium (LD).

### *k*-mer selection using *L*_1_ penalty and stability selection

Logistic regression is a widely used generalized linear model addressing binary classification problems. In our case, it consists in building a linear function defined for a strain represented by a vector *x*∈{0,1}^*p*^ as:

1 $$ f(x) = \beta_{0} + \sum\limits_{j=1}^{p} \beta_{j} x_{j},  $$

where *p* corresponds to the number of non-redundant *k*-mers obtained by the previous process, and *x* encodes the presence/absence of these *k*-mers. To estimate the model coefficients and simultaneously select a limited number of *k*-mers from a training panel of *n* strains, one can rely on the *L*_1_ or Lasso penalty and consider the following optimization problem:

2 $$ \hat{\beta} = \text{arg} \min_{\beta \in \mathbb{R}^{p+1}} \sum\limits_{i=1}^{n} L\left(y_{i}, f\left(x^{(i)}\right)\right) + \lambda \sum_{j=1}^{p} |\beta_{j}|,  $$

where *y*_*i*_=1 if the *i*th strain, represented by *x*^(*i*)^, is resistant and 0 otherwise. The function *L* is the logistic loss function, which quantifies the discrepancy between the true phenotypes *y*_*i*_ of the strains and the predictions *f*(*x*^(*i*)^) obtained by the model. The *λ* parameter achieves a trade-off between this empirical error and the Lasso regularization term, and is usually optimized by cross-validation.

Effectively tuning the Lasso regularization parameter is however a challenging problem. Cross-validation techniques usually succeed to build models of good predictive power, but the set of variables with non-zero coefficients is known to be unstable with respect to small variations in the training dataset or in the value of regularization parameter.

Several resampling based strategies have been proposed to cope with this issue [21, 23, 24]. We rely on the stability selection approach of [21], which is illustrated in Additional file 1: Figure S1. It consists in subsampling several times the entire dataset (step 1), solving for each subsample the Lasso-penalized logistic regression (step 2), and computing for each *k*-mer the proportion of models in which it was selected. This process is repeated for several values of the regularization parameter, which allows to define “stability paths” quantifying the probability of selecting each *k*-mer along the grid of regularization values (step 3). Instead of optimizing the regularization parameter *λ* itself, [21] propose to consider a threshold on the probability of being selected at some point on the regularization path: every *k*-mer whose stability path exceeds this threshold gets ultimately selected. Having identified stable *k*-mers, we finally fit a standard un-penalized logistic regression model that can be used to make predictions on new genome sequences (step 4). We note however that the issue of optimizing the regularization parameter of the Lasso is cast with this approach into that of optimizing the stability threshold, which can be done by cross-validation. To do so, we repeat the whole process described in Additional file 1: Figure S1 within each cross-validation fold, as detailed in “Models obtained” section. Using this optimized probability threshold, we repeat the process on the entire dataset to build the final model. In practice, we relied on the R software, and more precisely on the glmnet package [25], to compute regularization paths. Moreover, we considered subsamples involving half of the dataset and normalized each *k*-mer by its *L*_2_ norm to ensure an homogeneous level of penalization, as suggested and discussed in [21].

### Prediction from genome assembly or sequencing reads

Given sequencing data obtained from a new strain, the prediction process amounts to detecting the *k*-mers selected beforehand and making a prediction based on the score provided by the final logistic regression model, which can be turned into a probability of being resistant by the logistic function. We do not tolerate any mismatch to call a *k*-mer present and rely on the nucmer utility of the Mummer package [26] to do so. Predictions can be obtained from an assembled genome or directly from sequencing reads. In the latter case, however, a minimum threshold related to the sequencing depth must be considered to call a *k*-mer present from its number of occurrences, in order to be robust to sequencing errors.

Importantly, to call present each *k*-mer involved in a model, we rely on its entire set of equivalent *k*-mers. We detect each of them, and consider several strategies to ultimately call the *k*-mer present. The *stringent* strategy calls a *k*-mer present only if all its equivalent *k*-mers are detected. Conversely, the *conservative* strategy calls it present as soon as one of them is detected. Between these two possibilities, the *vote* strategy calls a *k*-mer present when more than half of its equivalent *k*-mers are detected, and the *smooth* strategy uses the proportion of equivalent *k*-mers detected instead of a binary presence/absence call. For instance, if we detected 8 *k*-mers out of a set of 10 equivalent ones, both the *conservative* and *vote* strategies would call the *k*-mer present, while the *stringent* would not. The *smooth* strategy would use a value of 0.8 – instead of 1 or 0 for presence or absence respectively – thereby effectively modulating the weight given to this *k*-mer by the logistic regression model, in order to account for the uncertainty in its detection. The optimal strategy to consider may in particular depend on the genomic plasticity of the bacterial species under study and the extent to which the training panel of strains properly accounts for it. Provided that the reads of the training data are available, it could easily be optimized by cross-validation techniques: the impact of the various strategies on the predictive performance could indeed be empirically measured and the best strategy retained. In this study, we solely assess the impact of the various strategies on the predictive performance, measured on independent validation sample, and leave the task of optimizing it automatically from the training data for future work. This prediction process takes a few seconds for an assembled genome and typically a couple of minutes for reads, depending on the sequencing depth (see “Predictive performance” section).

### Model interpretation

We aim to annotate the *k*-mers included in the model, to identify whether they fall within known genes or regulatory regions, and provide their putative function when possible. We consider the set of equivalent *k*-mers associated to each *k*-mer included in the model and try to reconstruct the longer stretch(es) of sequence(s) that they originate from. A set of equivalent *k*-mers usually corresponds to a larger sequence perfectly conserved across several strains of the panel, and sometimes to several such sequences in total LD. Following the terminology used by genome assembly algorithms, we assemble equivalent *k*-mers into longer *unitigs*, defined as the longest sequence(s) that can be obtained when they overlap by exactly *k*−1 nucleotides, using the bcalm2 software [27]. A unitig is interesting to represent a set of equivalent *k*-mers because it has the same presence/absence profile on the training genomes, but is in general longer that the individual equivalent *k*-mers, hence easier to annotate. Unitigs are finally aligned against one or several annotated reference genome(s) using the blastn-short program with a minimum of 80% identity and 85% coverage as filtering parameters, which, although not deeply optimized, gave satisfactory results in our experiments.

## Results and discussion

We now present a proof of concept of the method described in the previous section on two bacterial species, *M. tuberculolis* and *S. aureus*.

### *M. tuberculosis* study

#### Dataset constitution

We gathered two datasets from previous studies. The training dataset was taken from [19], who recently made available a set of 1306 assembled *M. tuberculosis* genomes, together with binary resistance phenotypes relating to 7 drugs, namely ethambutol, ethionamide, isoniazid, kanamycin, ofloxacin, rifampicin, and streptomycin. The resistance phenotype of each strain was not always available for each drug, but for each drug the number of resistant and susceptible strains was reasonably high (Table 1). The validation dataset involved 1586 strains^1^ that were previously used to validate the performance of the Mykrobe software [7]. Binary resistance phenotypes were available for 5 out of the 7 antibiotics included in the training panel, but not for ethionamide and ofloxacin. We used moxifloxacin as a proxy for ofloxacin resistance, since these two drugs belong to the same family and exhibit a very high level of cross-resistance [28]. We note that a variable number of each phenotype was available for each antibiotic, with in particular only nine strains resistant to kanamycin and ofloxacin (Table 2). This made estimating the sensitivity of the predictive models for these drugs uncertain. We also note that we directly worked from raw sequencing data to obtain predictions, that is, without relying on a prior step of genome assembly.

**Table 2:** *M. tuberculosis* study: validation results

		Stability		Mykrobe	
	R / S	sensi.	speci.	sensi.	speci.
ethambutol	194 / 1391	60.3 (6.9)	97.5 (0.8)	71.6 (6.3)	95.8 (1.1)
isoniazid	370 / 1216	89.7 (3.1)	97.5 (0.9)	84.3 (3.7)	98.6 (0.7)
kanamycin	9 / 460	33.3 (30.8)	98.9 (0.9)	33.3 (30.8)	99.6 (0.6)
ofloxacin	9 / 478	55.6 (32.5)	99.6 (0.6)	55.6 (32.5)	100 (0)
rifampicin	303 / 1262	94.1 (2.7)	99 (0.5)	93.7 (2.7)	99 (0.5)
streptomycin	353 / 1227	77.9 (4.3)	99.1 (0.5)	78.8 (4.3)	99.3 (0.5)

R / S: number of resistant and susceptible strains. Stability : sensitivity (sensi.) and specificity (speci.) values obtained with the stability-based final models. Mykrobe: sensitivity and specificity values obtained with the Mykrobe predictor. Figures into brackets correspond to half of the width of the 95% confidence intervals (CI) that shoud be added and substrated to get the upper and lower bounds of the 95% CI

A total of 19,876,230 distinct *k*-mers of length 31 were obtained from the 1306 training (assembled) genomes. 5,113,633 *k*-mers remained after filtering those occurring in less than three strains or in all the strains but one or two, which approximately resulted in a fourfold reduction. This set of *k*-mers corresponded to 151,403 sets of equivalent *k*-mers, among which a single one was randomly picked to define the set of non-redundant candidate variables to learn the models. This drastic reduction of the number of *k*-mers was due to the high clonality of *M. tuberculosis* genomes.

#### Models obtained

**Fig. 1:**
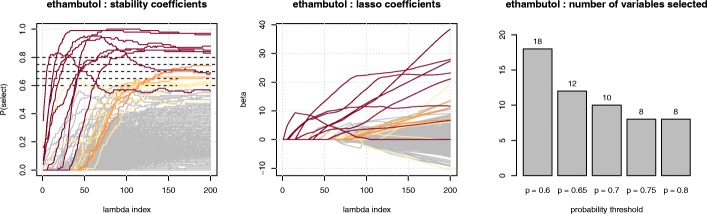
Illustration of the stability selection process for ethambutol. Left: stability paths. Each curve corresponds to a *k*-mer and represents its selection frequency over all the resampled datasets, across the values of the regularization paramater. Darker red curves correspond to larger selection probabilities (from 0.6 to 0.8), while grey curves correspond to *k*-mers with probability of selection below 0.6. Middle : the regularization path obtained by fitting a *L*_1_ penalized logistic regression model across the entire dataset, with *k*-mers colored according to the color code defined from the left panel. Right : number of *k*-mers selected by the stability selection approach for thresholds ranging from 0.6 to 0.8

As described in “*k*-mer selection using *L*_1_ penalty and stability selection” section and illustrated in Fig. 1, the sole parameter to optimize in our approach is the probability threshold of the stability selection procedure, which defines the set of stable *k*-mers. In this study, it was optimized by cross-validation over the grid {0.6;0.65;0.7;0.75;0.8}, for each antibiotic. Three repetitions of a 10-fold cross-validation process were carried out and the value of the parameter was chosen according to the average Area Under the (Receiver Operating Characteristic – ROC) Curve (AUC) obtained: the highest threshold allowing to reach the highest AUC value up to one point was retained, which allowed to favour sparser models for a comparable accuracy. The same cross-validation procedure was also applied to evaluate the standard *L*_1_-penalized logistic-regression approach. In both cases we considered a grid of 200 candidate values of the regularization parameter. It was defined by the glmnet software and ranged in a log scale from a maximum value defined as the smallest value ensuring that at least one variable is selected (i.e., has a non-null coefficient), to a minimum value defined as this maximum value divided by 10^4^. For the stability selection approach, we resampled 100 times the training dataset.

Table 1 summarizes the predictive performance and number of *k*-mers selected. As expected from [21], the stability procedure led to sparser signatures than the classical *L*_1_ penalization, especially for the ethambutol, kanamycin, and streptomycin antibiotics, for a slight decrease in terms of AUC. The predictive performance remained comparable, with the largest drop of 3 points observed for ethioniamide. Figure 1 illustrates the stability selection process for ethambutol. We noted in this example that some*k*-mers with a relatively high probability of being selected (e.g., ≥0.7, in orange), hence likely to be important to obtain accurate predictions, entered relatively late in the global regularization path of the Lasso, and vice-versa, which explained why the Lasso model involved many more *k*-mers. Additional file 1: Figures S2 and S3 show the same curves for the other antibiotics.

#### Predictive performance

We then evaluated the models obtained by stability-selection on the sequencing reads of the validation panel. We considered thresholds to call a *k*-mer present from its number of occurrences in reads ranging from 1 to 50, and the four strategies mentioned in “Prediction from genome assembly or sequencing reads” section to call a *k*-mer involved in a model present, based on the detection of its equivalent *k*-mers. While the threshold on the number of occurrences could be optimized for each sample, we systematically set it to 10. No major difference was observed as soon as it was not too low (e.g., 1 or 2 leading to false positive *k*-mer detection) or not too high (e.g., 25 or 50 missing some *k*-mers), as illustrated in Additional file 1: Figure S4. The differences observed between the various summarization strategies were also minor, and we systematically relied on the stringent approach.

**Fig. 2:**
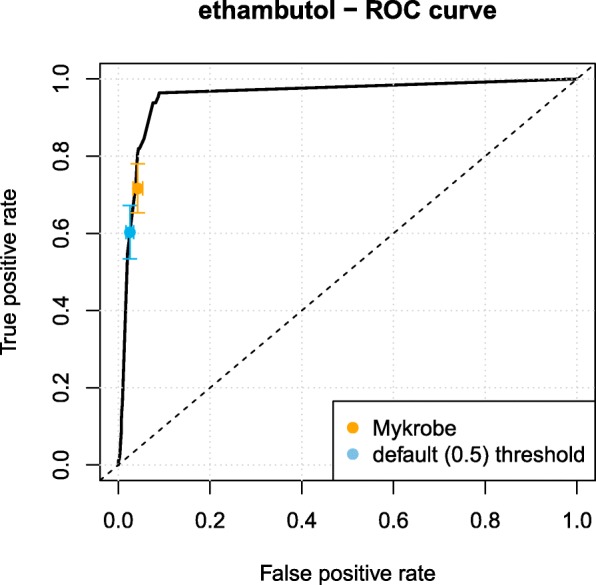
Ethambutol ROC curve obtained using the *k*-mers based signature evaluated in the validation dataset. The orange dot represents the performance obtained by the Mykrobe predictor, and the blue one to our *k*-mer based approach when using the default threshold of 0.5 to predict a strain resistant based on the probability provided by the logistic regression model. Arrows represent 95% confidence intervals

Table 2 showed that the performances obtained on the validation dataset by our approach and the Mykrobe predictor software were comparable. The most important differences were observed for the antibiotics ethambutol and isoniazid, where Mykrobe showed a higher and lower sensitivity, with an opposite effect on specificity. A ROC curve analysis indicated however that the sensitivity/specificity trade-offs achieved by Mykrobe could be met by modifying the decision threshold of the regression logistic model, which was set by default to 0.5. This is illustrated in Fig. 2 for ethambutol, and in Additional file 1: Figure S5 for the other antibiotics. The flexibility offered by the logistic regression model to control the trade-off between sensitivity and specificity can be useful in a diagnostics context to meet the target performance set by regulatory agencies.

We noted finally that the sequencing depth greatly varied in the validation set, with a minimum value around 20 and a maximum greater than 700. The prediction time increased linearly with the sequencing depth, showing that 1.25 s were necessary to process each coverage unit, which allowed to process a sample with a 100x coverage in about two minutes (Additional file 1: Figure S6).

#### Model interpretation

To interpret the predictive models obtained, sets of equivalent *k*-mers were assembled into unitigs, as described in “Model interpretation” section, and blasted against the H37Rv reference genome. The unitigs were highly conserved, with a coverage equal to 100% for all the unitigs and a minimum percent identify equal to 96.7% (rifampicin model, unitig #3). Each set of equivalent *k*-mers corresponded to a single unitig whose length ranged from 31 (the size of the individual *k*-mers) to 61 nucleotides, with a median value of 44.5 nucleotides (Additional file 1: Figure S7). These unitigs were easier to annotate than the individual *k*-mers because they were more specific of particular genomic regions, hence led to less ambiguous blast hits.

**Fig. 3:**
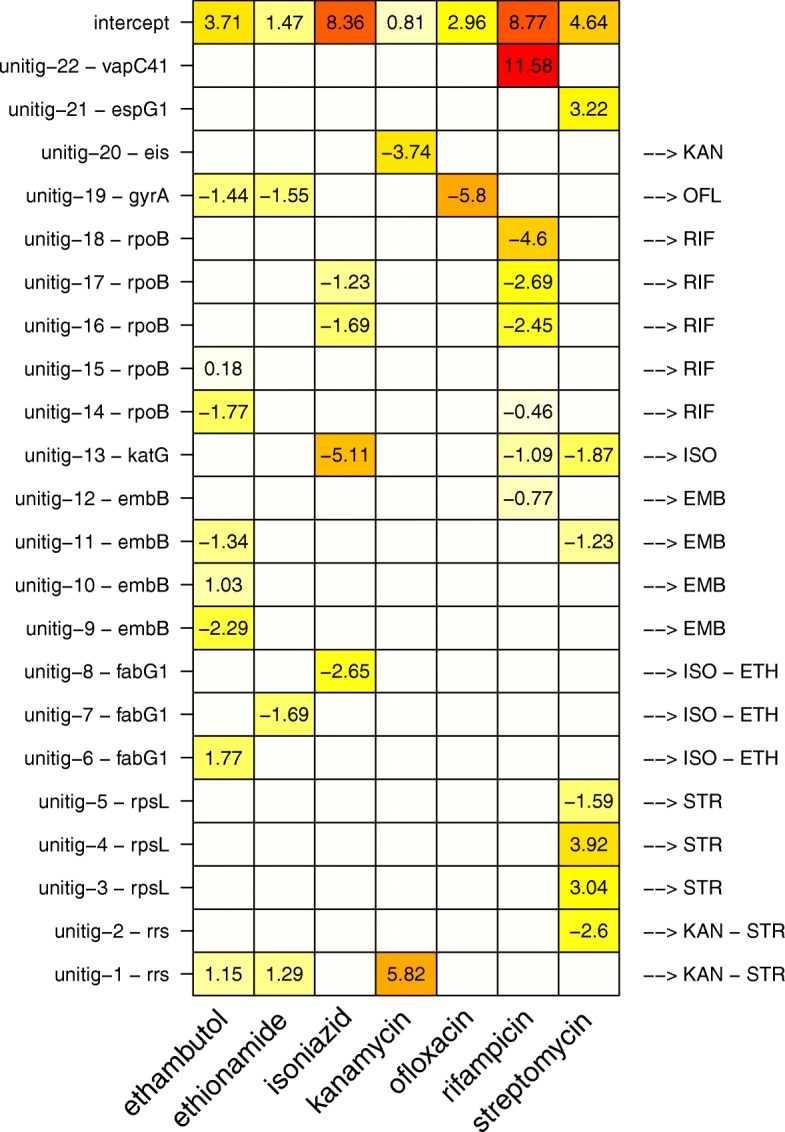
Signatures annotation at the unitig level. In total, 22 unitigs falling in 10 genes were retained. Known target / antibiotics association are shown on the right hand side of the figure. Figures correspond to *β* coefficients in the unpenalized final logistic model and colors to their magnitude (in absolute value). A negative coefficient leads to a decreased risk of resistance, *i.e.*, the presence of the corresponding unitig in the strain genome is associated with a decreased risk of antibiotic resistance, and conversely for a positive coefficient. A strain is predicted as resistant is the resulting score, taking into account the intercept of the model, is positive

Figure 3 represents the models and annotations obtained. Interestingly, without any any prior information of known resistance determinants, a total of 22 unitigs was retained (1 to 8 per antibiotic), relating to 10 genes or RNA often already known to be associated with *M. tuberculosis* antibiotic resistance, with unitigs originating from the *embB* gene for ethambutol, *fabG1* for ethionamide, *katG* and *fabG1* for isoniazid, *rss* and *eis* for kanamycin, *gyrA* for ofloxacin, *rpoB* for rifampicin, and *rss* and *rpsL* for streptomycin [12, 29]. Note that the *fabG1* gene is located just before the *inhA* gene, which is one of the two main markers of resistance to isoniazid and ethionamide. Some mutations associated to resistance said to originate from the promoter region of *inhA*, as for instance in [9], could actually be considered to fall within the *fabG1* gene.

We noted that some *k*-mers were part of in several signatures. This was probably due in large part to the level of correlation between resistance phenotypes observed within the training panel (Additional file 1: Figure S8). While in some cases such correlation is expected, for instance between rifampicin and isoniazid or between ethionamide and isoniazid [12], it may also be a consequence of a peculiar constitution of the training dataset due to a sampling bias, as discussed in “Conclusion” section.

Interestingly, two *k*-mers involved in four signatures originated from the 16S RNA (*rrs*). While this could be expected for kanamycin and streptomycin [12], we noted also that these *k*-mers captured general population or “clade” effects, as illustrated in Additional file 1: Figure S9. This suggested that resistance may in some cases be intrinsically related to the evolutionary history of the strains, which makes identifying causal resistance determinants difficult [17].

**Fig. 4:**
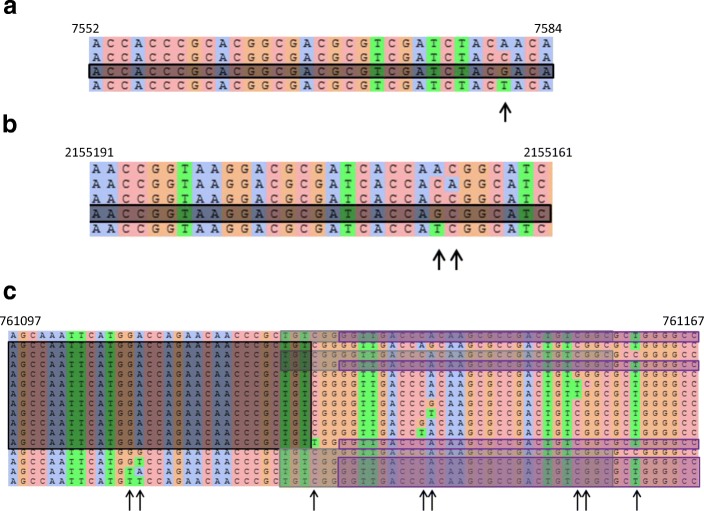
Examples of genomic variation patterns captured by *k*-mers in the ofloxacin, isoniazid, and rifampicin signatures. For each signature, mutliple alignments of haplotypes found in the training dataset are shown. Unitigs are surrounded by colored boxes and coordinates refer to nucleic positions on the H37Rv chromosome (NC_000962.3). **a** Ofloxacin - DNA gyrase subunit A - gene gyrA: a single SNP in *gyrA* predicts oxfloxacin resistance. At the SNP position, the 4 bases can be observed in the training dataset, the haplotype with the “G” being wild-type sensitive phenotype. **b** Isoniazid - catalase peroxidase - gene katG: 2 SNPs in *katG* predict isoniazid resistance, and these 2 SNPs are captured by a single unitig. **c** Rifampicin - DNA directed RNA polymerase beta subunit - gene rpoB: 8 SNPs in *rpoB* predict rifampicin resistance, and these 8 SNPs are captured by 3 unitigs

A striking observation was that the predictive models involved much fewer genetic determinants than alternative approaches relying on catalogues of mutations. For instance, [9] compiled a library of 1345 mutations used to predict the resistance to 15 antibiotics. Narrowing the list to the 7 antibiotics considered in this study led to a list of 745 mutations, much greater than the number of *k*-mers obtained by our approach. There are at least two reasons for that. First, the library of mutations was obtained by compiling results of several studies, involving altogether probably a much greater number of strains than used here. Reproducing our approach on a more exhaustive dataset would most likely lead to selecting more *k*-mers. However, we noted that *k*-mers allowed to represent in a concise way complex patterns of genetic variations, as illustrated in Fig. 4 for 3 genes included in the ofloxacin, isoniazid, and rifampicin signatures. These graphs were obtained by mapping the unitigs corresponding to the *k*-mers of the signatures against the training genomes, extracting and aligning the unique hits obtained and eventually representing the unitigs on the aligned haplotypes. In the upper panel, the unitig falling in the *gyrA* gene (obtained from 3 equivalent *k*-mers) captured a single SNP: a nucleotide other than “G” at the SNP position increased the risk of resistance to ofloxacin. In this simple example, 1 *k*-mer (or equivalently 1 unitig) indeed corresponded to a single mutation. It appears at position 7581 of the H37Rv chromosome and has already been widely described [9]. In the middle and lower panels, however, unitigs captured more complex resistance determinant patterns than a single SNP. The middle panel showed that a single unitig captured the increased risk of resistance to isoniazid brought by two distinct well known SNPs [9], occurring at positions 2155167 and 2155168 in the *katG* gene, and defining 5 distinct haplotypes. The genomic variability observed in the *rpoB* gene in the lower panel was even more complex. We noted indeed that the unitigs corresponding to three *k*-mers of the signature fell in the same region of the gene. Eight SNPs were observed in the training panel in this region between positions 761109 and 761161. They fall in the well-known rifampicin resistance-determining region [12], and all of them have been described in [9], except that observed at position 761156. Using only 3 *k*-mers, the model could therefore account for a much greater number of SNPs combinations, these eight SNPs defining 16 haplotypes in the training panel.

We note finally that the model coefficients allowed to measure the relative importance of the various *k*-mers defining the signatures. These coefficients could indeed be interpreted as odds ratio, a well known statistical indicator measuring the strength of association between a variable and an outcome. We emphasize also that they defined, through the logistic regression model, a probability of resistance, a positive coefficient indicating a higher risk of resistance. This property was interesting because it allowed us to associate a level of confidence to the prediction, and to control the trade-off between sensitivity and specificity that can be achieved by the model (see “Predictive performance” section).

### *S. aureus* study

We applied the same procedure to *S. aureus*. The training dataset was taken from [6]. It involved 501 genomes and 6 antibiotics, namely ciprofloxacin, erythromycin, fusidic acid, methicillin, penicillin and tetracyclin. Three other antibiotics were not considered here because the number of resistant strains was too limited (7 and 3 out of 501 for rifampicin and gentamicin respectively, and 2 out of 176 for mupirocin). The validation dataset included 470 genomes and phenotypes, and was also used in [7] to demonstrate the performance of the Mykrobe predictor software. Additional file 1: Table S1 gives the number of resistant and susceptible strains for these 6 antibiotics.

Table 3 summarizes the results obtained. We first noted that the stability selection approach also led to very sparse models (1 to 5 *k*-mers per model), often sparser than the classical Lasso. Predictive performance was in general comparable or slightly lower than that obtained by Mykrobe, except for fusidic acid where our model significantly lacked sensitivity. This was also the case, to a lesser extent, for ciprofloxacin where a drop of almost 6 points was observed, which corresponded to a difference of 4 strains out of 65 resistant ones and was not significant, as can be seen from the associated confidence intervals. We noted an effect of the strategy used to call present a *k*-mer involved in a signature from its set of equivalent *k*-mers. We observed indeed that while the stringent strategy was appropriate in most cases, better results could be obtained for penicillin using the smooth strategy and for tetracyclin using either the vote or the conservative strategy. Additional file 1: Figure S10 shows the impact of the various strategies, as well as the threshold on the number of *k*-mer occurrences, on the predictive performance. Table 3 gives the best result obtained for each antibiotic, with a threshold set to ten.

**Table 3:** *S. aureus* study: validation results

	Stability			Mykrobe	
	*k*-mers	sensi.	speci.	sensi.	speci.
ciprofloxacin	1 (18)	89.2 (7.5)	99.8 (0.5)	95.4 (5.1)	99.8 (0.5)
erythromycin	3 (8)	96.2 (4.2)	99.5 (0.7)	98.7 (2.5)	100 (0)
fusidic acid	3 (26)	78 (12.7)	100 (0)	100 (0)	99.1 (0)
methicillin	1 (1)	98.1 (3.6)	100 (0)	100 (0)	100 (0)
penicillin	1 (1)	99.7 (0.5)	88.3 (6.5)	99.7 (0.5)	88.3 (6.5)
tetracyclin	5 (7)	100 (0)	99.8 (0.4)	100 (0)	99.8 (0.4)

R / S: number of R/S strains. Stability: sensitivity (sensi.) and specificity (speci.) values obtained with the stability-based final models. Mykrobe: sensitivity and specificity values obtained with the Mykrobe predictor. Figures into brackets correspond to half of the width of the 95% confidence intervals (CI) that shoud be added and substrated to get the upper and lower bound of the 95% CI

Finally, Additional file 1: Table S2 presents the annotations of the unitigs obtained. Starting without a priori from more than 18 million *k*-mers, reduced to 335.238 filtered and non-redundant ones, known resistance determinants were identified for all drugs. We noted however that sets of equivalent *k*-mers were sometimes assembled into several unitigs, which therefore corresponded to non contiguous stretches of sequences in total LD within the training dataset. This was in particular the case for penicillin and tetracyclin, which may explain why the stringent *k*-mer summarization strategy was not appropriate for these antibiotics.

## Conclusion

We applied a machine learning approach to predict bacterial resistance phenotypes, starting from their whole genome sequences and without any prior information about the underlying resistance determinants. Using a penalized logistic regression model, coupled with a stability selection approach, we obtained predictive models involving a very limited number of *k*-mers, yet allowing to reach a performance comparable to alternative state of the art bioinformatics strategies for two bacterial species. The *k*-mers obtained uncovered previously known resistance determinants, thereby confirming that such a data driven strategy is promising to unravel bacterial genotype-phenotype relationships [18, 19]. The approach is generic and could readily be transposed to new bacterial species, and/or phenotypes, provided that adequate training data is available.

By selecting them in a discriminative fashion, our data-driven strategy allows to build complex prediction rules from a limited number of *k*-mers, as shown here for *M. tuberculosis*. A potential drawback of this approach, however, is that it intrinsically relies on the level of information provided by the training dataset. In particular, if the level of genomic variability around a causal determinant (e.g., a SNP) is under-represented in the training data because of a sampling bias, our approach will lead us to build too large sets of equivalent *k*-mers, that will not be detected as such in a new strain showing a different genomic context around the determinant. The various strategies proposed to summarize equivalent *k*-mers may therefore be useful to cope with this issue. Moreover, the study led on *M. tuberculosis* revealed that this approach is sensitive to the level of correlation between phenotypes. Some *k*-mers capturing resistance determinants within the target of a given antibiotic were involved in the model predicting resistance to another antibiotic, simply because strains of the training panels tended to be resistant to both antibiotics. *M. tuberculosis* strains may be simultaneously resistant to several antibiotics, which is in part due to the fact that therapies involve antibiotic “cocktails” [13]. In a predictive context where correlation between antibiotics is a biological reality, we consider that such correlation patterns may actually be helpful and leveraged by the model. The overlap we observed between signatures may actually capture some synergistic effects driving simultaneously the resistance to several antibiotics. Coll et al. [9] showed that specific mutations tended to co-occur among multi-drug resistant strains, and evidence has been reported on other bacterial species that a mutation conferring resistance to a given antibiotic could also increase the level of resistance to other antibiotics [30]. This observation also suggests, however, that explicitly learning jointly these predictive models within a multi-task learning framework may be a promising way to exploit such correlation patterns. Several extensions of the Lasso have been proposed to enforce tasks to share a common support, depending on their level of correlation [31]. They could provide an interesting way to study and leverage such cross-resistance mechanisms. If however this correlation is specific to the training dataset, hence results from a sampling bias, it can clearly compromise the generalization of the model. Drouin et al. [18] and Davis et al. [19] proposed different strategies to compensate for this correlation while learning predictive models, relying respectively on a subsampling of the dataset, or a post-processing of the list of *k*-mers selected. Within the penalized regression framework considered here, an alternative strategy could be to rely on multi-task appproaches enforcing tasks to have disjoint supports [32]. More generally, correlation between phenotypes is an issue to establish causal relationships between genetic determinants and antibiotic resistance. It represents a confounding factor for bacterial genome-wide association studies that should be taken into account, as it is commonplace for population structure [17].

The versatile framework of the penalized logistic regression offers many perspectives to further investigate bacterial genotypes/phenotypes relationships. Besides the extensions to the multi-task setting mentioned above to model cross-resistance mechanisms, it can easily be extended to consider semi-quantitative measurement of antibiotic resistance, using the MIC as outcome for an ordinal regression model [33]. It is indeed known that mutations can sometimes induce a variable level of resistance [12], and working directly from MICs may lead to better models. Likewise, correlation between strains due to their underlying population structure should be taken into account, by reducing the loss incurred by close strains or relying on mixed-model strategies [34].

In terms of diagnostics, the ability to carry out the prediction from reads coupled with the emergence of nanopore technologies paves the way to real-time sequencing-based applications [35]. A recent proof of concept, led on *M. tuberculosis* and starting from direct respiratory samples, demonstrated the feasibility of this approach [36]. How such *k*-mer based strategies could be transposed to metagenomics settings, in order to predict resistance directly from a sample, with a higher level of sequencing noise, remains an open question and will be the purpose of future work.

## Additional files

Additional file 1 Supplementary information and figures. (PDF 2415 kb)

Additional file 2 Summary of the training dataset involved in the *M. tuberculosis* study. (CSV 189 kb)

Additional file 3 Summary of the test dataset involved in the *M. tuberculosis* study. (CSV 352 kb)

Additional file 4 Summary of the dataset (training and test) involved in the *S. aureus* study. (CSV 213 kb)

## Abbreviations

AUC: Area under the (ROC) curve
LD: Linkage disequilibrium
MIC: Minimum inhibitory concentration
NGS: Next-generation sequencing
ROC: Receiver operating characteristic
SNP: Single-nucleotide polymorphism

## Footnotes

1 The original panel involved 1609 strains but 23 of them have missing phenotypes for all the drugs considered in this study.

**Table 1:** *M. tuberculosis* study: cross-validation results

	R / S	k-mers	AUC	*p*
ethambutol	333 / 712	8 (79)	91.0 (92.0)	0.75
ethioniamide	172 / 250	3 (9)	82.2 (85.3)	0.7
isoniazid	815 / 472	4 (5)	96.2 (96.0)	0.8
kanamycin	187 / 484	2 (49)	90.1 (93.2)	0.8
ofloxacin	238 / 458	1 (1)	91.2 (91.1)	0.8
rifampicin	668 / 533	7 (6)	96.6 (96.6)	0.8
streptomycin	492 / 678	7 (22)	90.8 (92.3)	0.8

R / S: number of resistant and susceptible strains. *k*-mers: number of *k*-mers in final models obtained with the stability selection approach and with the classical lasso penalty (between brackets). AUC: cross-validated AUC obtained with the stability selection approach and with the classical lasso penalty (within brackets). *p*: probability threshold selected for the stability approach
