# Supplementary material for: Predicting bacterial resistance from whole-genome sequences using k-mers and stability selection
Source: BMC Bioinformatics. 2018 Oct 17;19:383. doi: 10.1186/s12859-018-2403-z (PMC6192184; doi:10.1186/s12859-018-2403-z)
Supplement: Supplementary file 1 — Supplementary information and figures. (PDF 2415 kb) [file 12859_2018_2403_MOESM1_ESM.pdf]

Predicting bacterial resistance from whole-genome sequences using k-mers and stability selection - Supplementary materials

Pierre Mahé, Maud Tournoud

August 28, 2018

Contents

S1 Stability selection process 2

S2 *Mycobacterium tuberculosis* study 2

S2.1 Stability selection curves 2
S2.2 Prediction process 3
S2.3 ROC curve 3
S2.4 Prediction time 5
S2.5 Length of unitigs 7
S2.6 Phenotypes correlation 7
S2.7 16s kmers 8

S3 *Staphylococcus aureus* study 8

S3.1 Dataset constitution 8
S3.2 Prediction process 9
S3.3 Unitigs annotation 10

S4 Datasets 13

S1 Stability selection process

Figure S1 provides further details about the stability selection process.

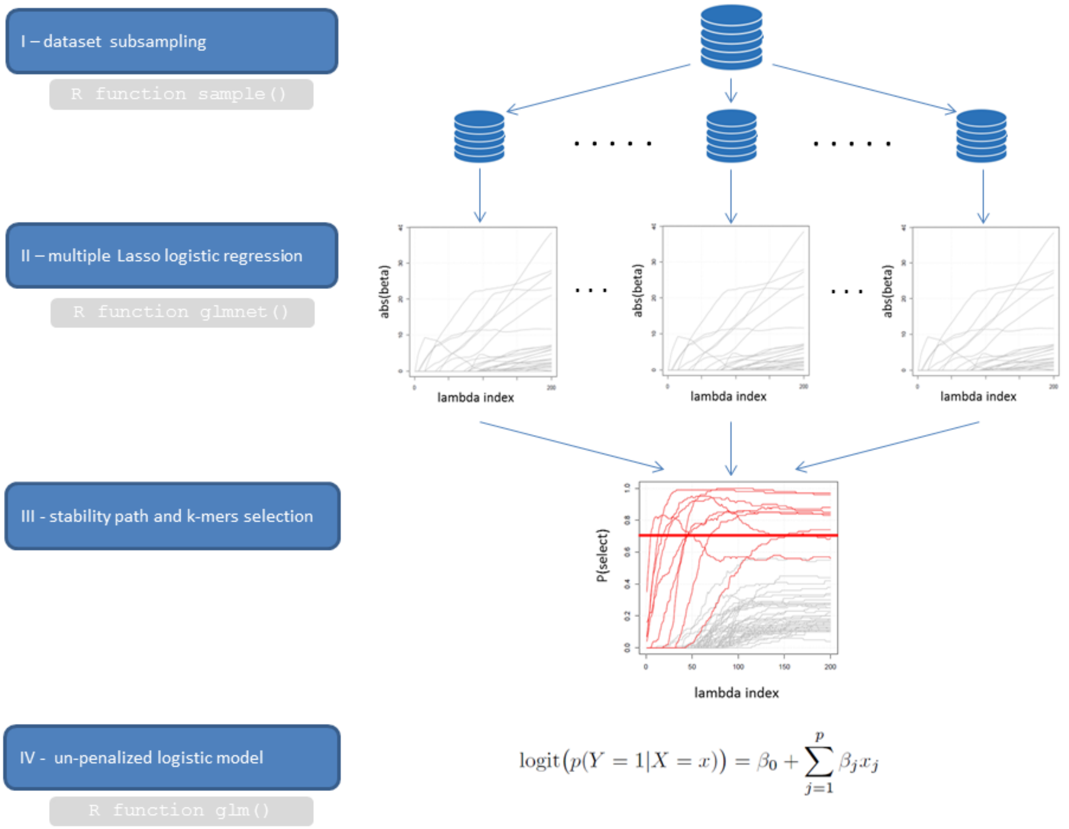

Figure S1: **Stability selection process**. In a first step, we create 100 random subsampled datasets including each 50% of the total number of resistant and susceptible strains, using the `sample()` function of `R`. For each subsampled dataset, we then fit a Lasso logistic model with a grid of 200 candidate values of the regularization parameter, using the `glmnet()` function of the `glmnet` package. This allows to define a regularization path per subsampled dataset, providing for each k-mer the value of the corresponding model coefficient (shown here on the y axis and on absolute value), according the value of the regularization parameter (shown on the x axis). The 100 regularization paths are then used to build a stability path for each k-mer : for each value of the regularization parameter, we retrieve the fraction of subsampled datasets in which the k-mer is selected by the lasso (i.e., in which it has a non-null coefficient). This defines the probability of selecting the k-mer along the grid of regularization values. We finally fit an un-penalized logistic regression model including the k-mers whose selection probability exceeds at some point a specified threshold. This last step is performed using the `glm()` function of `R`.

S2 *Mycobacterium tuberculosis* study

S2.1 Stability selection curves

Figures S2 and S3 show the stability paths obtained for the 7 antibiotics of the *M. tuberculosis* study. The probability ultimately selected is optimized by cross-validation.

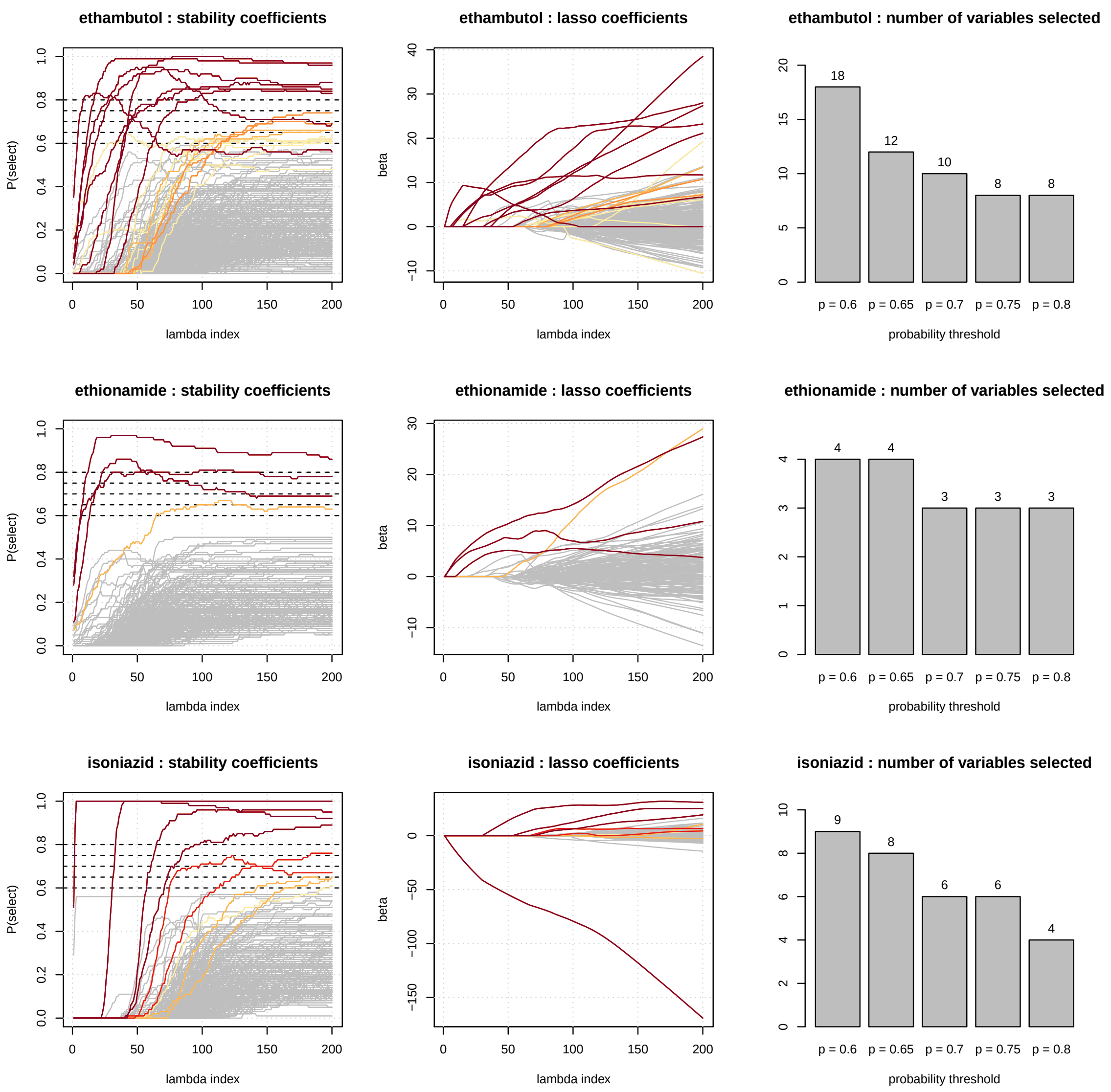

Figure S2: *M. tuberculosis* study: stability selection process - 1/2

S2.2 Prediction process

Figure S4 illustrates the process of calling k-mers present based on the detection of their equivalent k-mers, according to the procedure described in Section 2.3 of the main document. These figures represent the AUC obtained on the test dataset for the various configurations considered, that is (i) varying the threshold to call an equivalent k-mer present between 1 and 50, and (ii) summarizing them into a single variable to be used by the model according to the 4 strategies considered (stringent, conservative, vote and smooth).

S2.3 ROC curve

Figure S5 shows the ROC curves obtained (on the test dataset) for the various antibiotics with our k-mer based logistic regression approach. The threshold on the probability provided by the regression model to call a strain resistant was varied from its default 0.5 value to reach different sensitivity/specificity trade-offs. The values obtained by the default threshold are shown in blue

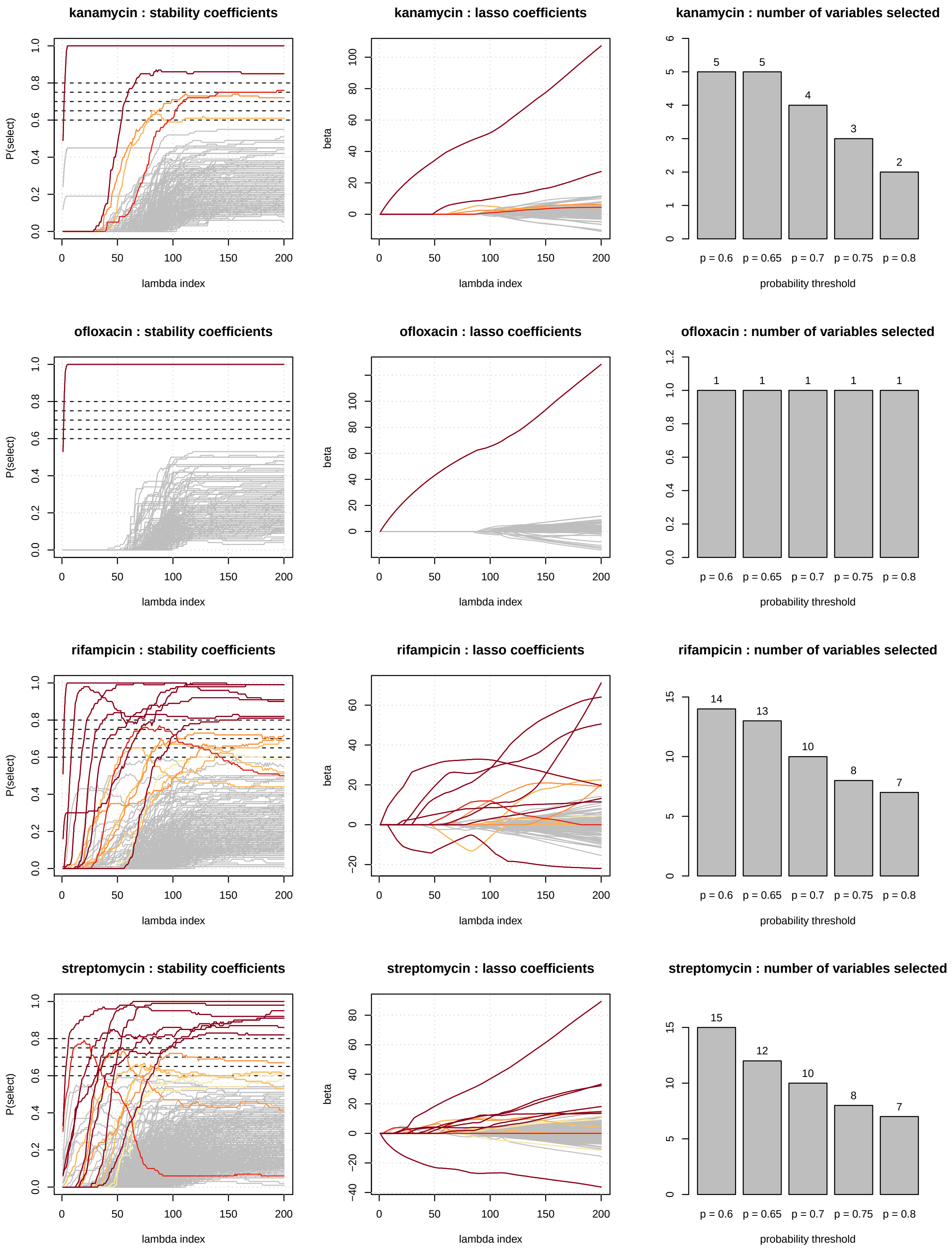

Figure S3: *M. tuberculosis* study: stability selection process - 2/2

and that obtained by Mykrobe in orange.

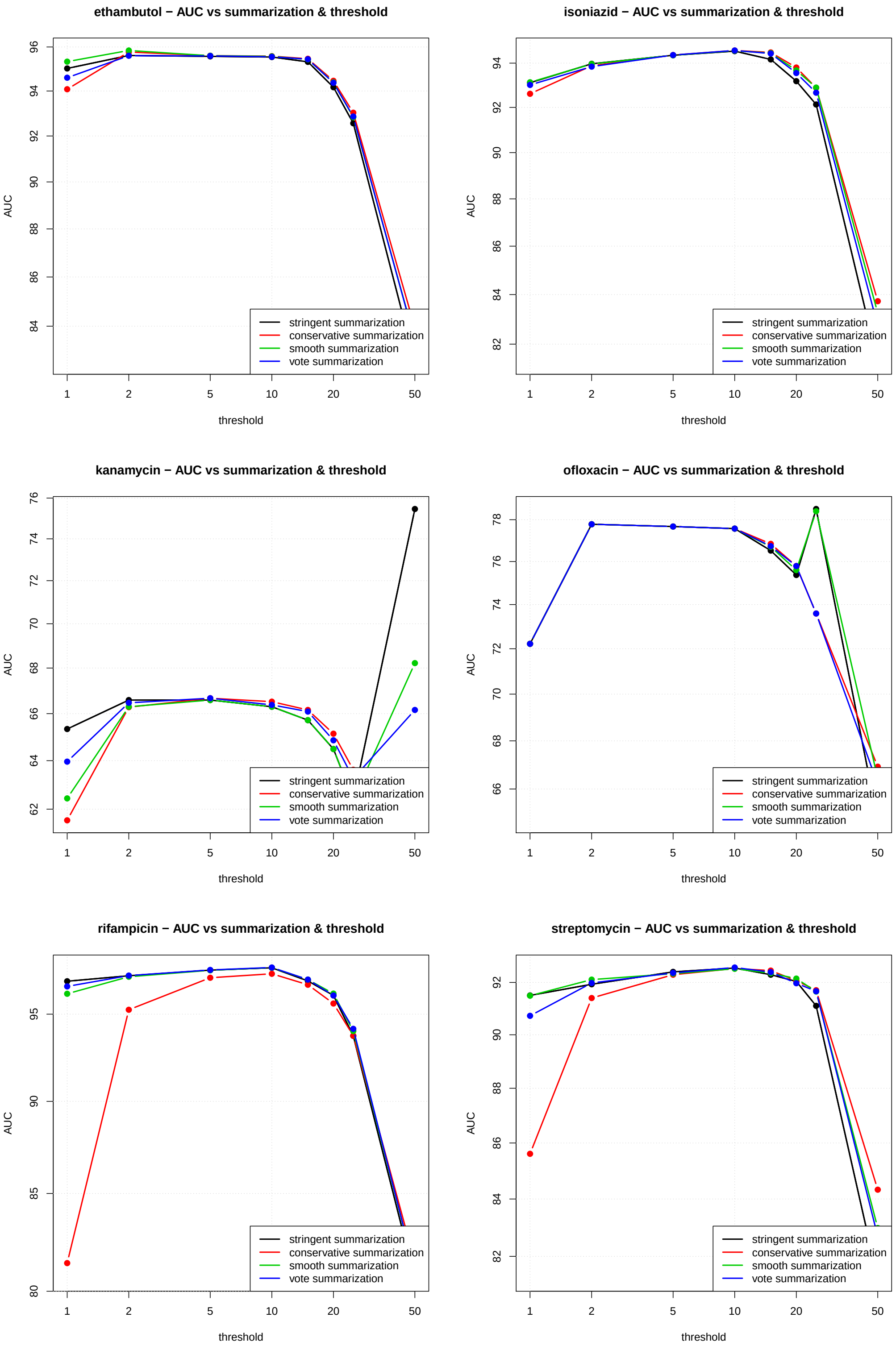

Figure S4: Illustration of the prediciopn process of calling k-mer present based on their equivalent k-mers.

S2.4 Prediction time

Figure S6 shows the prediction time measured to analyze reads datasets of various sizes. We noted indeed that the sequencing depth greatly varied in the validation set, with a minimum value

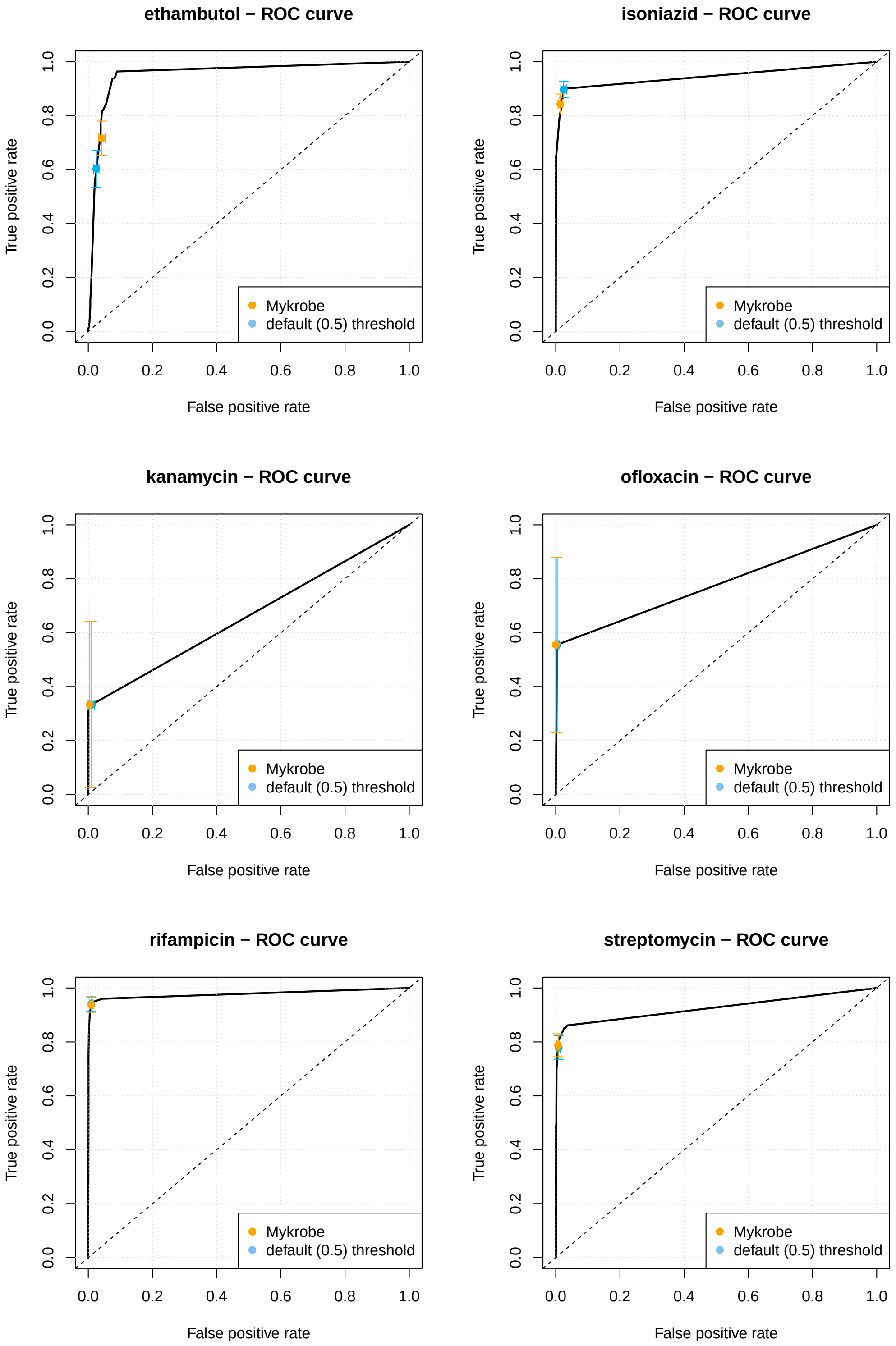

Figure S5: ROC curves obtained using k-mers based logistic regression models for the 6 antibiotics evaluated in the Mykrobe dataset. The orange dots correspond to the Mykrobe predictor and the blue ones correspond to our kmers-based models. Arrows represent 95% confidence intervals.

of nearly 11 and a maximum greater than 350. The prediction time increased linearly with the sequencing depth, with a slope of 1.25, that is, 1.25 second was necessary to process each coverage unit. We note however from this graph that this value constitutes an upper bound for samples of moderate coverage (less than 50 to 75). We note also that the prediction time corresponded to detecting the presence of 433 k-mers (obtained by merging the 22 sets of equivalent k-mers) in the 1st set of paired reads, i.e., only half of the sequencing data available.

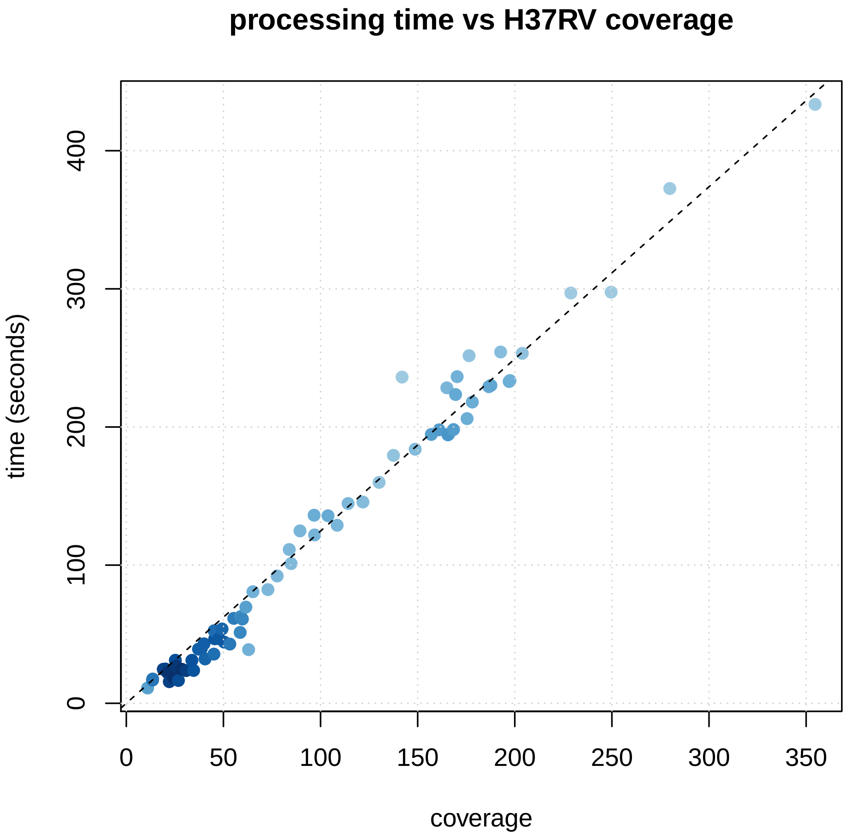

Figure S6: Prediction time vs sequencing coverage (relative to the H37RV genome).

S2.5 Length of unitigs

Figure S7 presents the length in bp of the unitigs obtained from equivalent k-mers included in the *Mycobacterium tuberculosis* signatures. Length of unitigs range from 31 bp to 61 bp, with a median value of 44.5 bp across all the signatures. Working with unitigs instead of k-mers makes the annotation easier, because longer sequences are more specific than shorter ones.

S2.6 Phenotypes correlation

Figure S8 presents the level of correlation between resistance phenotypes in the training (PATRIC) and validation (Mykrobe) panels. We observe in both datasets a strong correlation between the first-line drugs: isoniazid and rifampicin phenotypes in both datasets, and between isoniazid, rifampicin, streptomycin, and ethambutol in the validation dataset. We also observe, to a lesser extent, a correlation between ethionamide and ethambutol phenotypes in the training dataset. It is

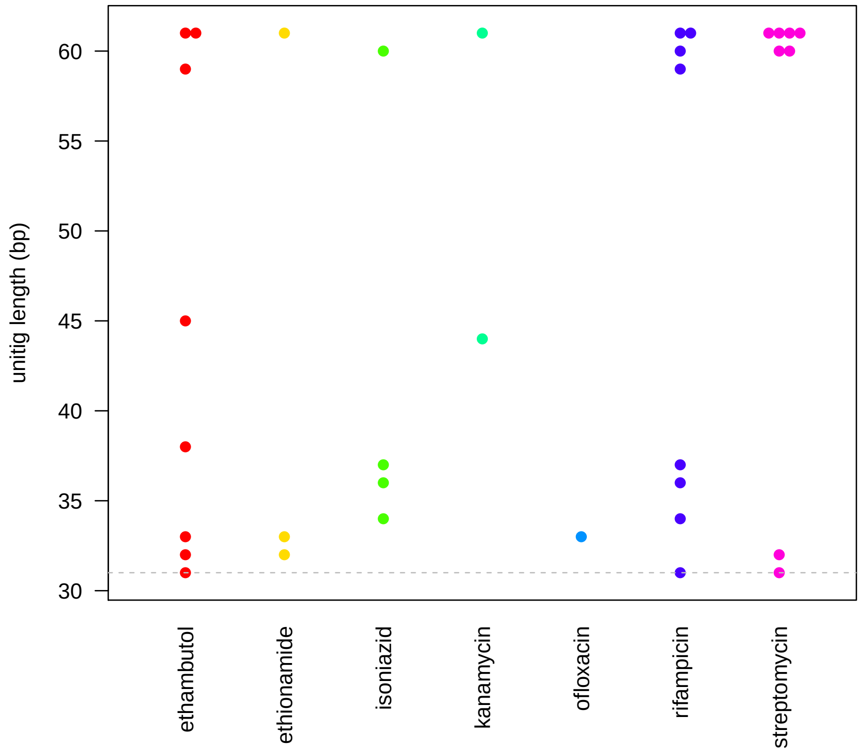

Figure S7: Length of the unitigs (in bp) obtained for the final *Mycobacterium tuberculosis* signatures. Each color corresponds to an antibiotic.

interesting to see that although these 2 datasets are independent, we observe very similar patterns of correlations.

S2.7 16s kmers

Figure S9 ilustrates the clade effects captured by the two k-mers found in the 16s rRNA gene. These figures were generated by the ItoL software[1].

S3 *Staphylococcus aureus* study

S3.1 Dataset constitution

Table S1 gives the constitution of the *S. aureus* dataset. The training dataset includes 501 genomes and resistance phenotypes for 6 antibiotics used in used in Gordon *et al.* (2014) and the validation dataset includes 470 genomes and phenotypes used in Bradley *et al.* (2015). The initial validation data from Gordon *et al.* (2014) included 490 genomes, but were excluded by Bradley *et al.* (2015) because of contamination issues.

[1] `http://itol.embl.de/`

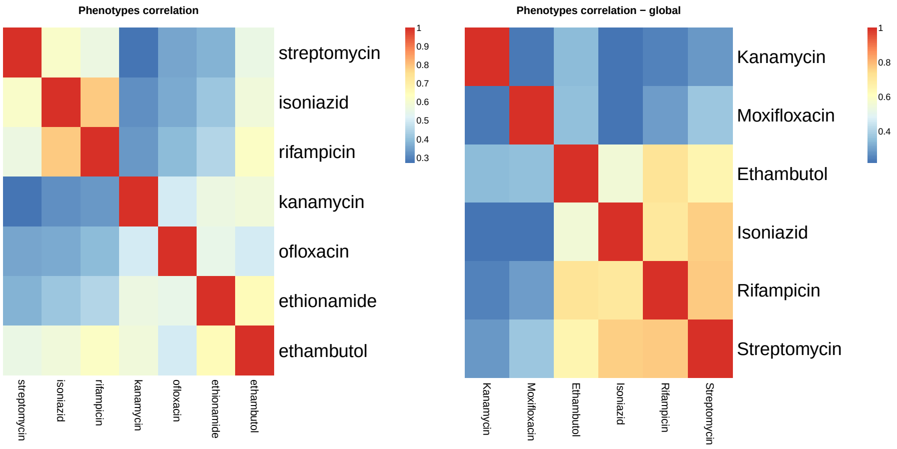

Figure S8: Phenotypes correlation - PATRIC (Left) and Mykrobe (Right) panels.

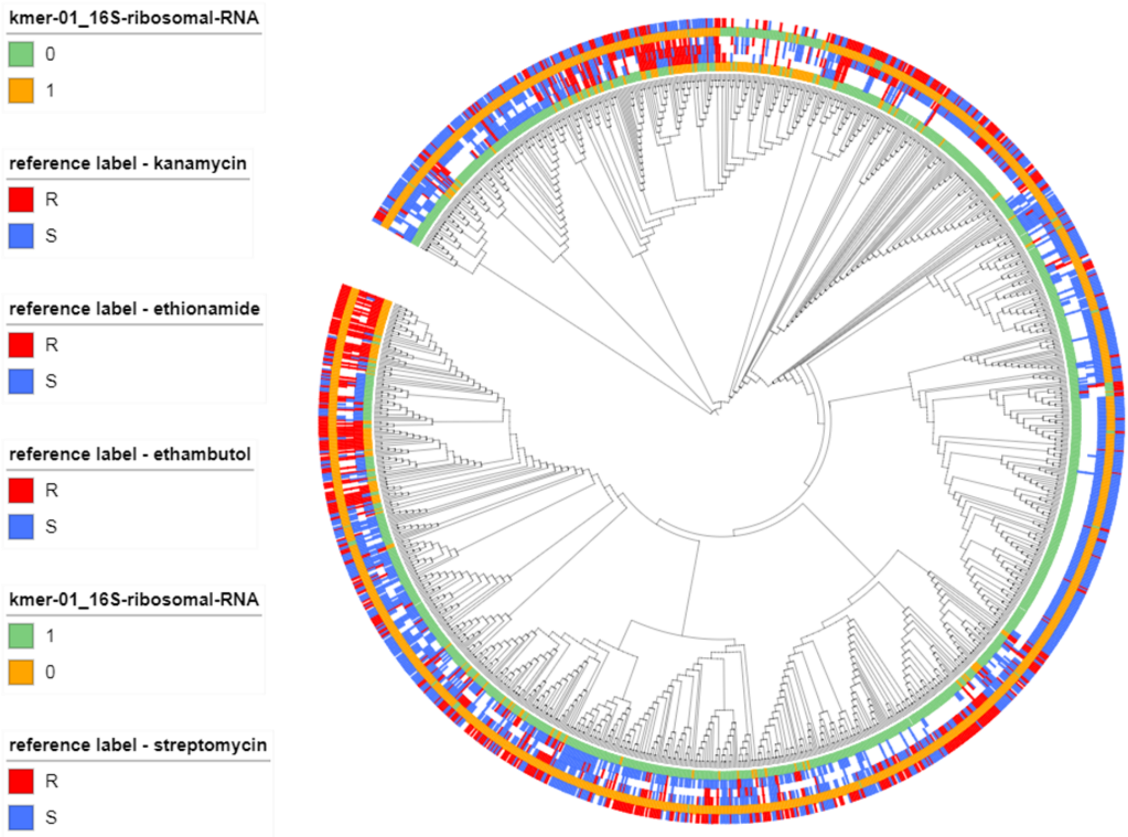

Figure S9: 16s k-mers. This figure represents a phylogeny of the training dataset, obtained from the k-mer based representation of strains. The annotations shown around the phylogeny are in the order of the legend shown on the left-hand side, and represent the presence/absence profiles of the two k-mers falling in the 16s gene, together with the (reference) phenotypes of the drugs that involve them in their predictive models.

S3.2 Prediction process

Figure S10 illustrates the process of calling k-mers present based on the detection of their equivalent k-mers, according to the procedure described in Section 2.3 of the main document, and as

	Training		Validation	
	R	S	R	S
cipropflaxin	172	329	65	405
erythromycin	134	367	79	391
fusidic acid	41	460	41	429
methicillin	159	342	54	416
penicillin	442	59	376	94
tetracyclin	28	473	17	453

Table S1: *S. aureus* study: training dataset used in Gordon *et al.* (2014) and validation dataset used in Bradley *et al.* (2015). Each column presents the number of Resistant and Susceptible strains with available genomes and phenotypes.

shown in Figure S4 for *M. tuberculosis*. While the various configurations considered had little influence on *M. tuberculosis*, we noted that the "stringent" strategy was clearly not optimal for Tetracycline, and to a lesser extent for Penicillin. Results reported in the main document are those obtained with the threshold set to ten, and the best summarization for each antibitioc (that is "stringent" for all but Penicillin, for which we used the "smooth" strategy, and Tetracycline, for which we used the "conservative" one).

S3.3 Unitigs annotation

Table S2 provides the annotations of the unitigs obtained in the *S. aureus* study. A similar procedure to that used for *M. tuberculosis* was applied. Sets of equivalent k-mers were first assembled into unitigs, which were then mapped to annotated reference genomes. The first important difference that can be noticed, is that contrary to what was observed previously, a single set of equivalent k-mers could lead to several unitigs. This is for instance the case of Penicillin, where a single set of equivalent k-mers led to 4 unitigs, and means that this set of equivalent k-mers corresponded to 4 non-contiguous stretches of sequence in total linkage disequilibrium in the training panel. We also note that the annotations are sometimes less straightforward than for *M. tuberculosis*, with several unitigs mapping to hypothetical proteins in particular.

	kmer-id	unitig-id	annotation	position
Ciprofloxacin	1	1	DNA gyrase subunit A	within-CDS
Methicillin	1	1	Methicillin resistance protein MecR1	within-CDS
Penicillin	1	1	beta-lactamase (Penicillinase)	within-CDS
		2	regulatory protein blaR1	within-CDS
		3	beta-lactamase (Penicillinase)	within-CDS
		4	beta-lactamase (Penicillinase)	within-CDS
Fusidic acid	1	2	fusidic acid resistance protein	within-CDS
		3	hypothetical protein	promoter
		4	fusidic acid resistance protein	within-CDS
		5	hypothetical protein	promoter
		6	hypothetical protein	promoter
		7	hypothetical protein	promoter
		8	hypothetical protein	within-CDS
		9	fusidic acid resistance protein	within-CDS
		10	no annotation	
		11	hypothetical protein	within-CDS
		12	hypothetical protein	promoter
	2	1	elongation factor G (FusA gene)	within-CDS
	3	1	elongation factor G (FusA gene)	within-CDS
Erythromicin	1	1	transposase C for Tn554	promoter
	2	2	rRNA adenine N-6-methyltransferase (ermC gene)	within-CDS
	3	3	no annotation	
Tetracycline	1	1	TetM	within-CDS
		3	hypothetical protein (similar to Tn916 in E faecalis)	within-CDS
		4	transposase	promoter
		6	TetM	within-CDS
		9	TetM	within-CDS
		11	transcriptional regulator	within-CDS
		12	TetM	within-CDS
		13	hypothetical protein (similar to Tn916 in E faecalis)	within-CDS
		14	TetM	within-CDS
		16	hypothetical protein (similar to Tn916 in S pneuomoniae)	within-CDS
		17	hypothetical protein	within-CDS
		18	transposase from transposon (Integrase)	within-CDS
		19	hypothetical protein (similar to Tn916 in S pneuomoniae)	within-CDS
		22	hypothetical protein (similar to Tn916 in E faecalis)	within-CDS
		24	TetM	within-CDS
		25	hypothetical protein (similar to Tn916 in E faecalis)	within-CDS
		26	TetM	within-CDS
	2	23	hypothetical protein (similar to Tn916 in E faecalis)	within-CDS
	3	2	TetM	promoter
		5	hypothetical protein (similar to Tn916 in E faecalis)	within-CDS
		7	hypothetical protein (similar to Tn916 in E faecalis)	within-CDS
		8	TetB	promoter
		20	transposase from transposon (Integrase)	promoter
	4	21	TetB	within-CDS
	5	15	TetB	within-CDS

Table S2: *S. aureus* study : annotation of the unitigs obtained. "k-mer id" : index of the k-mer in the predictive models. "unitig-id" : index of the unitig(s) obtained by assembling the corresponding set of equivalent k-mers.

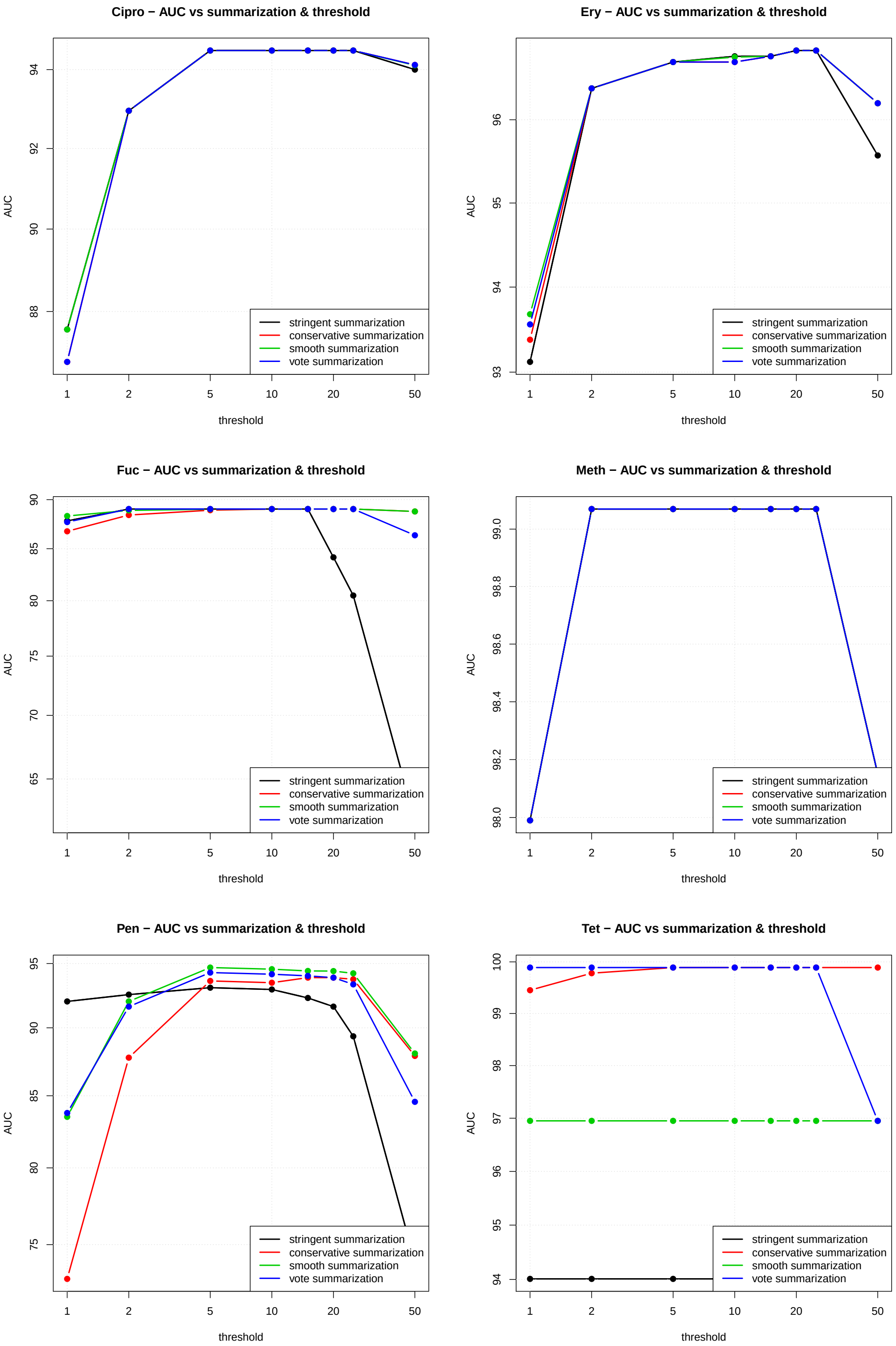

Figure S10: Illustration of the predictiopn process of calling k-mer present based on their equivalent k-mers.

S4 Datasets

All the datasets used in this study have been involved in previous works and are available in public repositories. The following files, available as supplementary materials of this paper, provide links to sequence data and reference phenotyes:

- *M. tuberculosis study*:
  - the file `TB_training.csv` provides a summary of the training dataset, which was involved in Davis *et al.* (2016). Sequence data are assembled genomes, which are available on `PATRIC`[2].
  - the file `TB_test.csv` provides a summary of the test dataset, which was involved in Bradley *et al.* (2015). Sequence data are raw reads, which are available on `SRA`.
- *S. aureus study*: the file `SA_training-test.csv` provides a summary of the dataset (both training and test), which was involved in Bradley *et al.* (2015). Sequence data are raw reads, which are available on `SRA`. Note that this dataset was originally involved in Gordon *et al.* (2014). The initial validation data included 490 genomes, but were excluded by Bradley *et al.* (2015) because of contamination issues.

References

Bradley, P., Gordon, N. C., Walker, T. M., Dunn, L., Heys, S., Huang, B., Earle, S., Pankhurst, L. J., Anson, L., de Cesare, M., Piazza, P., Votintseva, A. A., Golubchik, T., Wilson, D. J., Wyllie, D. H., Diel, R., Niemann, S., Feuerriegel, S., Kohl, T. A., Ismail, N., Omar, S. V., Smith, E. G., Buck, D., McVean, G., Walker, A. S., Peto, T., Crook, D., and Iqbal, Z. (2015). Rapid antibiotic-resistance predictions from genome sequence data for *Staphylococcus aureus* and *Mycobacterium tuberculosis*. *Nature Communications*, **6**, 10063.

Davis, J. J., Boisvert, S., Brettin, T., Kenyon, R. W., Mao, C., Olson, R., Overbeek, R., Santerre, J., Shukla, M., Wattam, A. R., Will, R., Xia, F., and Stevens, R. (2016). Antimicrobial resistance prediction in PATRIC and RAST. *Scientific Reports*, **6**, 27930.

Gordon, N. C., Price, J. R., Cole, K., Everitt, R., Morgan, M., Finney, F., Kearns, A. M., Pichon, B., Young, B., Wilson, D. J., Llewelyn, M. J., Paul, J., Peto, T. E. A., Crook, D. W., Walker, A. S., and Golubchika, T. (2014). Prediction of *Staphylococcus aureus* Antimicrobial Resistance by Whole-Genome Sequencing. *Journal of Clinical Microbiology*, **52**(4), 1182–1191.

[2] `ftp://ftp.patricbrc.org/patric2/current_release/AMR_genome_sets`
